# Sociodemographic and environmental factors associated with diarrhoeal illness in children under 5 years in Uganda, 2016: a cross-sectional study

**DOI:** 10.1186/s12879-023-08458-8

**Published:** 2023-07-18

**Authors:** Nathan Ssekandi, Nonhlanhla Tlotleng, Nisha Naicker

**Affiliations:** 1 Epidemiology and Surveillance Section, National Institute for Occupational Health, National Health Laboratory Services, 25 hospital St, Construction hill, Braamfontein, South Africa; 2 Department of Environmental Health, Faculty of Health Sciences, University of Johannesburg, Johannesburg, South Africa; 3 School of Health Systems and Public Health, University of Pretoria, Arcadia, South Africa

**Keywords:** Diarrhoea, Sociodemographic factors, Environmental factors, Children under five, Uganda

## Abstract

**Background:**

Uganda is among the 10 countries in the sub-Saharan Africa region that have the highest prevalence of diarrhoeal disease. Evidence suggests that the severity of childhood diarrhoeal disease is escalated through various sociodemographic and environmental factors.

**Objectives:**

To assess prevalence of diarrheal illness in children below the age of 5 years in Uganda in 2016 and associated factors.

**Methods:**

A cross-sectional study was employed that analyzed secondary data from the 2016 Uganda Demography and Health Surveys. Children with and without diarrhea were compared. A logistic regression was used to determine sociodemographic and environmental factors associated with diarrheal illness in children with statistical significance at p < 0.05.

**Results:**

The prevalence of childhood diarrhoeal illness for children below the age of 5 years in Uganda was 20.9% (n = 2838/13,753). There was a statistically significant difference when comparing children diarrhoeal with the following sociodemographic factors: caregiver’s age, child’s age and gender and duration of breastfeeding (p < 0.0001). Children with a caregiver aged between 15 and 24 years (aOR;1.42; 95% CI:1.24–1.62) and 25–34 years (aOR;1.19; 95% CI:1.04–1.37) were more likely to report diarrhoeal disease, compared to those with a caregiver aged 35–49 years. For environmental factors, households using springs water, access to health facility and children who received a dose of vitamin A had a decreased risk of reporting children diarrhoeal.

**Conclusion:**

Significant factors in the study like caregiver’s age, gender and duration of breastfeeding will create the opportunity for all interventions to shift their focus to these factors thus a better evidence-based approach to reducing of diarrhoeal disease will be achieved in the country.

## Background

Diarrhoeal disease is the passage of three or more watery stools per day, or the more frequent passage than normal for an average healthy person [1]. Diarrhoeal disease is linked to poor sanitation and hygiene, for example, defecating close to a water source or people forgetting to wash their hands; thus, transferring pathogens to their mouth in the process of eating [2]. The sub-Saharan Africa region and South Asia have the highest prevalence of diarrhoeal disease in the world, with countries like Congo, Sudan, India, Niger and Somalia topping the burden ranking [3]. Diarrhoeal disease in Uganda is among the top four causes of morbidity and mortality in children below the age of 5 years. The prevalence of diarrhoeal disease in Uganda in 2015 was 20% and the total deaths attributed to diarrhoeal disease increased from 5.0 to 6.4%, thus ranking the country 27th worldwide [4]. Currently, diarrhoeal disease is still among the top 10 causes of mortality among children, with most cases related to rotavirus, which causes up to 40% of all diarrhoeal cases in Uganda [4].

In Uganda, research has shown a strong association between diarrhoeal disease and risk factors including poverty, education, inappropriate feeding practices and unhygienic environmental conditions such as unprotected water sources [5]. In particular, poor sanitation remains a major problem affecting Ugandans, leading to the increased spread of diarrhoeal diseases. Over 30% of households in the country have unimproved toilets and practise open defecation; more than half a million households do not have toilet facilities; and 750,000 people do not have access to clean water [4].

The Wash and Sanitation Hygiene (WASH) interventions have been helpful to control the incidence of diarrhoeal disease and related morbidity and mortality in children. However, only 45% of people in Uganda have access to basic sanitation and only 63.2% of people are supplied with safe water [6]. Various studies on diarrhoea and the associated factors have been conducted in the country, with most in community and hospital settings. Investigating the causes of diarrhoeal disease is vital for designing effective intervention programs, informing policies, allocating resources and intervention prioritisation. Thus, this study was conducted to identify the factors associated with diarrhoeal disease among children under the age of 5 in Uganda. No study utilising data from the Uganda Demographic Health Survey (UDHS) 2016 to investigate factors linked with childhood diarrhoeal disease has been done.

## Materials and methods

### Study setting and design

The study setting was Uganda, located in East Africa. The population is estimated at 47.32 million people. Uganda is divided into 4 main administration regions which are dived into 15 sub-regions and consist of 112 districts. Over 74.45% of the country population stay in rural areas and 25.55% in urban areas. Rural areas of the districts are further subdivided into 146 sub-counties, parishes and villages. The country’s economy is based on agriculture sector through the production is mainly from livestock and crops [7]. Secondary data analysis was conducted from the primary data collected in the cross-sectional Ugandan Demographics and Health Survey (UDHS).

### Study population

The study population included children under the age of 5 years residing in 15 regions (South Central, North Central, Busoga, Kampala, Lango, Acholi, Tooro, Bunyoro, Bukedi, Bugisu, Karamoja, Teso, Kigezi, Ankole, and West Nile) in Uganda and had caretakers aged between 15 and 49 years (the age range the DHS used in their survey). Caretakers were interviewed about the child’s diarrhoeal illness. The caretaker was either the child’s father, mother or relative with adequate information on the child, the household features and resided in one of the 15 regions of Uganda.

### Sampling approach

The primary study was conducted by the Uganda Demography and Health Survey that used Uganda Bureau of Statistic (UBOS) to collect data. It used a stratified, two-stage cluster design in the sampling of the 20,880 selected households that comprised women and men, came from urban or rural areas in the 15 regions of Uganda. The sampling strategy excluded institutional groups like schools, army barracks, clinics, and police stations, and only included 15 regions, divided into 112 districts as clusters [7]. In the original study, 19,088 women aged 15–49 years, 5,676 men aged 15–49 years and 14,493 children aged 0–59 months were included [7]. Out of the 19,088 women, 18,506 completed the interview, which yielded a 97% response rate and for men out of 5,676 participants, only 5,336 participated successfully in the interview, which led to a response rate of 94% [7]. This study applied an inclusion and exclusion criteria which led to 13,573 participants in the final study sample size. In the secondary analyses, all data from the eligible participants (13,573) were analysed.

### Data collection

Uganda Bureau Of Statistic (UBOS) were the primary data collector. Standardized questionnaires on paper were used to collect data between to June 2016- December 2016 and then entered into Computer-assisted personal interviewing system (CAPI). The questionnaires were divided into four types, which included a women’s questionnaire, a household questionnaire, a men’s questionnaire, and the Biomarker questionnaire. These questionnaires reflected the health issues of the population important to Ugandan Government. Questionnaires were also translated into the eight major languages of Uganda: Ateso, Ngakarimojong, Luganda, Lugbara, Luo, Runyankole-Rukiga, Runyoro-Rutoro and Lusoga. Data on background characteristics (age, education, media exposure), reproduction, family planning, maternal and child health, breastfeeding, marriage and sexual activity, fertility preferences, STIs & HIV/AIDS, adult & maternal mortality, domestic violence, early childhood development, and other health issues was collected [7].

### Data analysis

Data was analysed using a Stata Software package version 17. College Station, TX;StataCorp LLC 2019. Descriptive statistics were carried out to understand the distribution of sociodemographic and environmental factors for the study. Frequencies and percentages were obtained for categorical variables. The prevalence of diarrhoeal disease was calculated, and independent variables described. Pearson’s Chi square was used to compare characteristics of those with and without diarrhoea. Bivariable and multivariable logistic regression models with Odds Ratios were used to examine the relationship of sociodemographic and environmental variables and diarrhoeal disease. From the unadjusted or crude model, variables with p-values < 0.2 were included in the adjusted logistic regression model. A backward stepwise regression model was applied to select variables used in the final regression model. A p-value of < 0.05 was the cut-off for statistically significant measures in the final model, with a 95% confidence interval.

## Results

Of the 13,573 caregiver-child pairs who responded to the diarrhoeal question, the prevalence of self-reported diarrhoea over the previous two weeks was 20.9% (n = 2838) (Table 1). The majority of the caregivers were in the age group 25–34 years (46.4%), with most having attained primary school education (62.6%) as the highest educational level. About 86.5% (n = 11,744) of the caregivers were married and 79.8% (n = 10,827) were unemployed. Most of the caregivers that were heads of a household were male (75.8%, n = 10,289). The majority of children in the study were below the age of 1 year (22.5%), with most being male (50.1%, n = 6801). The majority of the participants resided in rural areas (82.6%, n = 11,209), with many coming from the Western region (33.3%, n = 4514). While the majority (85.5%) have no TV, no electricity (75.3%) and no car (94.6%), most reported having a radio (53.4%). Most of the caregivers reported that children’s stools were properly disposed of (94.9%) where there was a latrine available (86.7%). Majority of the households did not share toilets (58.2%) and used pit latrines (84.6%). Water from wells was the commonest source (58.1%). The majority of caregivers visited a health facility (80.5%) and received diarrhoea medication (60.0%) for their children. The majority of the children in the study received a Vitamin A dose (66.0%) but did not get a rotavirus vaccine (91.9%).

**Table 1:** Prevalence of childhood diarrhoeal disease by socioeconomic and demographic characteristics of respondents in Uganda, UDHS (2016) [N = 13,573]

Characteristics	No diarrhoeal (n = 10,735) %	Had Diarrhoeal (n = 2838) %	*P*-value ^
**Caregiver’s age in years**			
15–24	3244 (73.7)	1160 (26.3)	0.000*
25–34	5076 (80.7)	1218 (19.4)	
35–49	2415 (84.0)	460 (16.0)	
**Caregiver’s education**			
No education	1501 (80.8)	356 (19.2)	0.069
Primary	6687 (78.7)	1809 (21.3)	
Secondary	1957 (78.4)	538 (21.6)	
Higher	590 (81.4)	135 (18.6)	
**Caregiver’s Martial status**			
Single	390 (76.3)	121 (23.7)	0.154
Married	9317 (79.3)	2427 (20.7)	
Divorced/separated/widowed	1028 (78.0)	290 (22.0)	
**Residence**			
Urban	1918 (81.1)	446 (18.9)	0.007*
Rural	8817 (78.7)	2392 (21.3)	
**Region**			
Central	2984 (77.2)	880 (22.8)	0.000*
East	1421 (82.8)	296 (17.2)	
North	2595 (74.6)	883 (25.4)	
West	3735 (82.7)	779 (17.3)	
**Caregiver’s employment status**			
Yes	8576 (72.2)	587 (21.4)	0.500
No	2159 (78.6)	2251 (20.8)	
**Caregiver’s sex (Household head)**			
Male	8173 (79.4)	2116 (20.6)	0.082
Female	2562 (78.0)	722 (22.00)	
**Religion**			
Catholic	7695 (79.2)	2016 (20.8)	0.791
Muslim	1310 (78.1)	367 (21.9)	
Protestant	1576 (79.1)	416 (20.9)	
Others	137 (80.6)	33 (19.4)	
No religion	17 (73.9)	6 (26.1)	
**Age of child (years)**			
0	2137 (70.0)	918 (30.1)	0.000*
1	1922 (68.6)	879 (31.4)	
2	2115 (79.9)	531 (20.1)	
3	2245 (88.1)	302 (11.9)	
4	2316 (91.8)	208 (7.3	
**Child’s sex**			
Male	5291 (77.8)	1510 (22.2)	0.000**
Female	5444 (80.4)	1328 (19.6)	
**Number of children < 5 in a household**			
Up to 2,	8105 (78.9)	2173 (21.1)	0.238
3, and more	2630 (79.8)	665 (20.2)	
**Duration of breastfeeding**			
Never	233 (86.9)	35 (13.1)	0.000*
Still breastfeeding	3432 (69.0)	1542 (31.0)	
Ever breastfeeding	7070 (84.9)	1261 (15.1)	
**Wealth index**			
Lowest	2843 (77.3)	836 (22.7)	0.001*
Second	2375 (78.6)	647 (21.4)	
Middle	2092 (79.5)	541 (20.6)	
Fourth	1787 (79.8)	452 (20.2)	
Highest	1638 (81.9)	362 (18.1)	
**Internet use**			
Yes	558 (79.6)	143 (20.4)	0.733
No	10,177 (79.1)	2695 (20.9)	
**Housing status**			
Sand	7536 (78.7)	2044 (21.3)	0.075
Wood	55 (76.4)	17 (23.6)	
Concrete	2843 (80.5)	687 (19.5)	
Others	301 (77.0)	90 (23.0)	
**Number of family members**			
1–9 households	9525 (79.0)	2526 (21.0)	0.630
10–18 households	1185 (79.6)	303 (20.4)	
19–27 households	25 (73.5)	9 (26.5)	
**TV**			
Yes	1335 (85.2)	290 (17.9)	0.004*
No	9133 (78.7)	2467 (21.3)	
**Radio**			
Yes	5865 (80.9)	1382 (19.1)	0.000*
No	4603(77.0)	1375 (23.0)	
**Car**			
Yes	321(84.5)	59 (15.5)	0.019*
No	10,147(79.0)	2698 (21.00)	
**Electricity**			
Yes	2419 (80.5)	587 (19.5)	0.071
No	8049 (78.8)	2170 (21.2)	

*^ Chi square test x* ^*2*^

*- Significant at 0.05

%- No of diarrhoea cases/total population*10

Childhood diarrhoea prevalence was significantly different depending on caregiver’s age, residence, northern rural region, child’s age, child’s gender, duration of breastfeeding, wealth index, ownership of a TV, radio and a car (Table 1). Diarrhoeal occurrence among children was significantly different in families that had two children < 5 years (21.1%), did not have access to internet (20.9%), house constructed of wood (23.6%), number of family members between 10 and 18 in household (79.6%) and did not have electricity (21.2%), had the highest prevalence of childhood diarrhoea. Table 2 shows the prevalence of childhood diarrhoeal disease with selected environmental characteristics. Latrine availability, families that shared a toilet, whose toilet type was either flush, pit or none, with source of water that was either piped, a well, springs, containers, visited a health facility and the child having received the vitamin A and a rotavirus vaccine were significantly different in terms of the occurrence of childhood diarrhoeal illness.

**Table 2:** Prevalence of childhood diarrhoeal disease and environmental characteristics of respondents in Uganda, UDHS (2016) [N = 13,573]

Characteristics	No diarrhoea (n = 10,735) %	Had diarrhoea (n = 2838) %	*P*-value ^
**Child stool disposal**			
Improper	339 (74.0)	119 (26.0)	0.250
Proper	6562 (76.4)	2031 (23.6)	
**Latrine availability**			
Yes	9382 (79.7)	2386 (20.3)	0.000*
No	1086 (74.5)	371 (25.5)	
**Toilet sharing**			
Yes	3710 (77.8)	1062 (22.3)	0.000*
No	5770 (81.0)	1356 (19.0)	
**Toilet type**			
Flush	241 (85.5)	41 (14.5)	0.000*
Pit	9141 (79.6)	2345 (20.4)	
None	1086 (74.5)	371 (25.4)	
**Source of water**			
Piped	1679 (80.3)	411 (19.7)	0.000*
Wells	6159 (78.1)	1728 (21.9)	
Springs	2545 (81.2)	590 (18.8)	
Containers (Packed)	62 (83.8)	12 (16.2)	
Others	23 (59.0)	16 (41.0)	
**Source of non-potable water**			
Protected	5 (71.4)	2 (28.6)	0.795
Unprotected	7 (77.8)	2 (22.2)	
Others	3 (100)	0	
**Visitation of health facility**			
Yes	8537 (78.1)	2392 (21.9)	0.000*
No	2198 (83.1)	446 (16.9)	
**Vitamin A dose**			
Yes	3954 (70.5)	1654 (29.5)	0.000*
No	2211 (76.7)	672 (23.3)	
**Rotavirus vaccine**			
Yes	424 (73.5)	153 (26.5)	0.759
No	5666 (72.5)	2147 (27.5)	

*^ Chi square test x* ^*2*^

*- Significant at 0.05

%- No of diarrhoea cases/total population*100

The unadjusted logistic regression analyses in Table 3 showed that children with caregivers between the ages of 15–24 years (cOR;1.64; 95% CI:1.49–1.81) and 25–34 years (cOR;1.21; 95% CI:1.10–1.33) were more likely to contract diarrhoeal disease, compared to those whose caregivers were aged 35–49 years. Children residing in rural areas were 1.13 times more likely to contract diarrhoeal disease, compared to those residents in urban areas (cOR:1.13; 95% CI:1.03–1.24). In addition, children from the Eastern and Western regions were less likely to suffer from diarrhoeal illness (cOR;0.76; 95% CI:0.67–0.85 & cOR;076; 95%CI:0.70–0.83) than children from the Northern region, who were more likely to suffer from diarrhoeal disease (cOR;1.11; 95%CI:1.03–1.21) compared to the Central region. Male children, under 3 years of age, were more likely to suffer from diarrhoeal illness, compared to children over the age of 4 years. Children from middle, second and lower wealth index families residing in houses built with sand were more likely to suffer from diarrhoeal disease.

**Table 3:** Bivariate and multivariate logistic regression analysis of sociodemographic & environmental factors associated with childhood diarrhoea in Uganda, UDHS (2016) [n = 13,573]

Characteristics	cOR (CI)	P-value	aOR (CI)	P-value
**Sociodemographic**				
**Caregiver’s age in years**				
35–49	1.00	-	1.00	-
15–24	1.64(1.49–1.81)	0.000*	0.84(0.77–0.92)	0.000*
25–34	1.21(1.10–1.33)	0.000*	0.71(0.62–0.81)	0.000*
**Caregiver’s education**				
Higher	1.00	-	1.00	-
No education	1.03(0.86–1.23)	0.749	0.95(0.82–1.11)	0.552
Primary	1.14(0.98–1.34)	0.095**	0.96(0.81–1.15)	0.665
Secondary	1.16(0.98–1.37)	0.090**	1.01(0.80–1.28)	0.940
**Caregiver’s Martial status**				
Married	1.00	-	1.00	-
Single	1.15(0.98–1.34)	0.095**	0.94(0.79–1.13)	0.559
Divorced/separated/widowed	1.06(0.95–1.18)	0.254	1.02(0.89–1.19)	0.722
Residence				
Urban	1.00	-	1.00	-
Rural	1.13(1.03–1.24)	0.000*	1.04(0.91–1.19)	0.554
**Region**				
Central	1.00	-	1.00	
East	0.76(0.67–0.85)	0.000*	0.65(0.56–0.75)	0.000*
North	1.11(1.03–1.21)	0.009*	0.96(0.85–1.08)	0.468
West	0.76(0.70–0.83)	0.000*	0.72(0.65–0.81)	0.000*
**Caregiver’s Employment status**				
No	1.00	-	- -	- -
Yes	0.97(0.90–1.05)	0.499		
**Caregiver’s sex (Household head)**				
Male	1.00	-	1.00	-
Female	1.07(0.99–1.15)	0.080**	1.01(0.92–1.12)	0.786
**Religion**				
No religion	1.00	-	-	-
Catholic	0.80(0.40–1.59)	0.516	-	-
Muslim	0.84(0.42–1.68)	0.620	- -	- -
Protestant	0.80(0.40–1.60)	0.529	-	-
Others	0.74(0.35–1.58)	0.442		
**Age of child (years)**				
4	1.00	-	1.00	-
0	3.65(3.17–4.20)	0.000*	-	-
1	3.81(3.31–4.39)	0.000*	1.02(0.93–1.12)	0.710
2	2.44(2.09–2.83)	0.000*	0.60(0.49–0.75)	0.000*
3	1.44(1.22–1.70)	0.000*	-	-
**Child’s sex**				
Female	1.00	-	1.00	-
Male	1.13(1.06–1.21)	0.000*	1.13(1.06–1.21)	0.002*
**Number of children < 5 in a household**				
Up to 2	1.00	-	1.00	-
3 and more	0.95(0.88–1.03)	0.240**	0.98(0.89–1.07)	0.620
**Duration of breastfeeding**				
Never breastfed	1.00	-	1.00	-
Ever breastfed	1.16(0.85–1.59)	0.356	1.05(0.69–1.61)	0.814
Still breastfeeding	2.37(1.74–3.24)	0.000*	1.33(0.89–2.03)	0.181
**Wealth index**				
Highest	1.00	-	1.00	-
Fourth	1.12(0.98–1.26)	0.085	1.10(0.89–1.35)	0.373
Middle	1.14(1.01–1.28)	0.038*	1.14(0.89–1.45)	0.296
Second	1.18(1.05–1.33)	0.004*	1.13(0.87–1.47)	0.349
Lowest	1.26(1.12–1.40)	0.000*	1.10(0.83–1.44)	0.501
**Internet use**				
No	1.00	-	-	-
Yes	0.97(0.84–1.13)	0.734	-	-
**Housing status**				
Concrete	1.00	-	1.00	-
Sand	1.09(1.01–1.18)	0.020*	1.06(0.92–1.23)	0.400
wood	1.21(0.80–1.85)	0.368	1.44(0.89–2.32)	0.139
Others	1.18(0.97–1.43)	0.089	1.05(0.55–1.99)	0.890
**Number of family members**				
1–9 households	1.00	-	-	-
10–18 households	0.97(0.87–1.08)	0.594	- -	- -
19–27 households	1.26(0.72–2.21)	0.415		
**TV**				
No	1.00	-	1.00	-
Yes	0.84(0.75–0.94)	0.002*	0.94(0.77–1.15)	0.565
**Radio**				
No	1.00	-	-	-
Yes	0.83(0.78–0.89)	0.000*	0.83(0.76–0.91)	0.000*
**Car**				
No	1.00	-	1.00	-
Yes	0.74(0.58–0.94)	0.012*	0.86(0.63–1.17)	0.340
**Electricity**				
No	1.00	-	1.00	-
Yes	0.92(0.85–1.00)	0.044*	1.01(0.88–1.16)	0.883
**Environmental**				
**Child stool disposal**				
Proper	1.00	-	1.00	-
Improper	109(0.94–1.29)	0.244**	0.97(0.79–1.19)	0.749
**Latrine availability**				
No	1.00	-	1.00	-
Yes	0.80(0.72–0.88)	0.000*	0.66(0.40–1.11)	0.115
**Toilet sharing**				
No	1.00	-	1.00	-
Yes	1.17(1.09–1.26)	0.000*	1.07(0.98–1.17)	0.107
Toilet type				
Flush Pit	1.00 1.40(1.06–1.87)	- 0.020*	1.00 1.34(0.91–1.96)	- 0.138
None	1.75(1.30–2.36)	0.000*	-	-
**Source of water**				
Piped	1.00	-	1.00	-
Wells	1.11(1.01–1.23)	0.028*	0.92(0.81–1.05)	0.207
Springs	0.96(0.85–1.07)	0.446	0.85(0.73–1.00)	0.031*
Containers (Packed)	0.82(0.49–1.39)	0.472	0.88(0.50–1.55)	0.649
Others	2.09(1.42–3.07)	0.000*	1.38(0.81–2.35)	0.231
**Source of non-potable water**				
Protected	1.00	-	-	-
Unprotected	-	-		
Others	-	-	-	-
**Visitation of health facility**				
No	1.00	-	1.00	-
Yes	1.30(1.18–1.42)	0.000*	1.20(1.30–1.35)	0.001*
**Vitamin A dose**				
No	1.00	-	1.00	-
Yes	1.27(1.17–1.37)	0.000*	1.42(1.30–1.57)	0.000*
**Rotavirus vaccine**				
No	1.00	-	-	-
Yes	0.96(0.84–1.11)	0.619	-	-

*cOR – Crude Odds ratio*

*aOR – Adjusted Odds ratio*

*- Significant at α = 0.05

*Ref -Reference variable*

** - Less than 0.2 were included in multivariate analysis

For environmental characteristics, the unadjusted regression analysis showed that latrine availability, toilet sharing, toilet type, source of water (piped & others), visiting a health facility and the vitamin A dose increased the risk of childhood diarrhoeal disease.

Adjusting the model with known covariates, sociodemographic characteristics: caregiver’s age, region (East & West), age of child (2 years), child’s gender, and owning a radio were significantly associated with reporting childhood diarrhoea. Children with a caregiver aged between 15 and 24 years (aOR;1.42; 95% CI:1.24–1.62) and 25–34 years (aOR;1.19; 95% CI:1.04–1.37) were more likely to contract diarrhoeal disease, compared to those with a caregiver aged 35–49 years. Children that came from the Eastern region (aOR;0.65; 95% CI:0.56–0.75) showed a reduced risk of 35% and from the Western region (aOR;072; 95%CI:0.65–0.81) a reduced risk of 28% to suffer from diarrhoeal illness, compared to those from the Central region. Children aged 2 years (aOR;0.60; 95% CI:0.49–0.75) showed a reduced risk of 40% of suffering from diarrhoeal disease, compared to those that were aged 4 years. Male children were more likely to suffer from diarrhoeal illness, compared to females (aOR;1.13; 95% CI:1.06–1.21). For environmental characteristics, source of water (springs), visitation to a health facility and getting a dose of vitamin A was significantly associated with childhood diarrhoeal disease. Children who received the vitamin A dose (aOR;1.42; 95% CI:1.30–1.57) were more likely to suffer from diarrhoeal disease, compared to those that had not received a vitamin A dose.

## Discussion

The study was able to identifying the factors that associated with burden of childhood diarrhoeal disease among children under age of 5 years in Uganda. Between Jaune to December 2016, the overall prevalence of diarrhoeal disease was 20.9% amongst children below the age of 5 years. Factors such as caregiver’s age, region (East or West), age of child and sex and having a radio were found to be significant factors associated with diarrhoeal disease. The results of this study were consistent with existing literature that assessed the prevalence of diarrhoeal illness in Uganda among children. Previous studies by Omona et al. (2020), Ocamanono (2018), Adeokun and Yaya (2020) and Nambuusi et al. (2020) reported diarrhoea prevalence among children in the range of 20–34%, with factors such gender, mother’s age and age of child found to either increase or decrease the occurrence of diarrhoeal illness [8–11]. Younger caregivers were associated with higher odds of diarrhoea in their children compared to older caregivers. Similar findings were found in a study conducted by Omona et al. (2020) and George et al. (2014). Older caregivers typically have more experience caring for children than their younger counterparts, which lowers the prevalence of childhood diarrhoea [8, 12].

Children over the age of two had a 40% reduced risk of suffering from diarrhoeal illness (aOR:0.60: 95% CI:0.49–0.75) compared to children aged 4 years. The study findings contradict other published research, where as a child grows older, he/she becomes less likely to experience diarrhoeal disease. Since younger children spend most of their time crawling on the floor and putting their unwashed fingers in the mouth, they are more likely to ingest contaminating microorganisms [13]. Education is key to combating childhood diarrhoeal disease. In the study, 13.7% of caregivers had no formal education and the majority (62.6%) attained only a primary education level in the study. Even though this was not a significant variable in the multivariate analysis, poorly educated people were more likely to be unaware of appropriate hygiene and sanitation measures thus, resulting into increased childhood diarrhoeal illness exposure [14].

Male children were more exposed to diarrhoeal disease (aOR:1.13: 95% CI:1.06–1.21), possibly due to malnutrition and high risk of exposure to diarrhoeal disease agent, compared to female children [15]. Similar findings were observed in research done by Paul (2020) in India and Sarker et al. (2016) in Bangladesh, who report that male children are more likely to experience a high prevalence of diarrhoeal illness [14, 16]. The higher prevalence may be due to the generally poor hygiene and sanitation among male children, since caretakers do not often take them for showers, compared to female children [14, 16].

Children who come from the Central region had a 35% reduced risk of suffering from childhood diarrhoeal illness (aOR;0.65; 95% CI:0.56–0.75) and those from the Western region an 18% reduced risk (aOR;072; 95%CI:0.65–0.81). Also, children who were two years old had a 40% reduced risk of suffering from diarrhoeal illness (aOR;0.60; 95% CI:0.49–0.75), compared to those who were 4 years old. A study done by Ssenyonga et al. (2017) in Uganda showed that the Eastern and Northern regions have the highest prevalence of diarrhoeal disease, compared to the Central region [17]. The Northern and Eastern regions have been plagued with rebel insurgencies and instability, which have hindered the regions progress in terms of establishing and advancing the healthcare systems [18]. In contrast, WASH interventions were successfully implemented in the Central and Western regions with collaboration from the community and external partners in 2015 [19].

In the assessment of the relationship between environmental factors and childhood diarrhoeal illness, only water source including using a well, visitation to a health facilities and having received a vitamin A dose, were significant factors associated with diarrhoeal illness. In the study, caregivers who used wells as a source of water had reduced odds of their children suffering from diarrhoeal illness, compared to those who use piped water (aOR;0.85; 95% CI:0.73–1.00). The findings of the study were consistent with a study done by Ssenyonga et al. (2017) in Uganda, and by Tumwine et al. (2002) in Mali [17, 20]. In Uganda, it is a traditional practise that all water collected from the well should either be boiled or treated prior to use. At the time of the study, only 15.4% of caregivers used piped water as source of water with the majority using protected wells. Unprotected wells are strongly linked with diarrhoeal illness due to most of the water being contaminated with the bacteria or parasites associated with diarrhoeal infection. However, most countries have started treating all water reservoirs through natural purification, photolysis and chemical water treatments [21].

In this study, caregivers who reported children with diarrhoeal illness indicated visiting a health facility (21.9%) and that the children had received a dose of vitamin A (29.5%). In regression analysis, caregivers who visited health care facilities and those whose children received a dose of vitamin A were more likely to report diarrhoeal illness in their children. Most children have only one dose of vitamin A, which may increase susceptibility to infectious diseases, specifically diarrhoeal diseases [22]. In addition, sick children attending the clinic will most likely get a vitamin A dose. Thus, the association with vitamin A may not be a direct link to diarrhoea but rather related to visiting a health facility. Also, rotavirus vaccine was not significant to the study since the coverage was low. The rotavirus vaccine was introduced into Uganda immunisation program in 2018 while the survey was conducted in 2016, so coverage figures likely to represent the private market. Child stool disposal was not found to be significant in the study; nonetheless, improper disposal could lead to increased diarrhoeal illness among children [23]. Latrine availability and toilet sharing were other factors that were not significant in the study. However, toilets are crucial structures in a house to prevent people from randomly defecating anywhere, which can result in the contamination of food and water when faeces are not properly cleared in the environment [24].

The study presents findings that add to the existing literature on the prevalence and risk factors of childhood diarrhoeal illness in Uganda. These findings indicate that additional prevention strategies to reduce the prevalence in Uganda may be necessary. Therefore, health practitioners’ engagement with the communities to improve education and training on potential risk factors of diarrhoeal illness as well as providing them with services to reduce disease may be needed. Although the study showed that education level was not a significant predictor of reducing or increasing childhood diarrhoeal illness, it is still advisable to raise awareness of diarrhoeal disease by encouraging caregivers to practise good hygiene. Other interventions could include creation of posters & charts that can be placed in the community or home as a reminder of diarrhoeal exposures and prevention approaches.

Future studies, using a mixed methods approach such as conducting community interviews to aid in better understanding, knowledge and perception of the community on risk factors of childhood diarrhoeal illness are recommended. Moreover, future research should seek to establish the extent to which diarrhoeal disease is unintentionally or accidentally acquired by children from different backgrounds, with such information assisting in developing target interventions. Also, with the introduction of rotavirus vaccine in 2018, there is need of more research on the coverage and its effects on the prevalence of diarrhoea and more encouragement of mothers accepting their children to go for rotavirus immunisation.

### Limitations

The study’s shortcoming included that the secondary dataset used in the analysis did not contain information on additional variables that could be factors or predictors of diarrhoeal illness. Crucial variables or confounders may have been omitted; their effect not accounted for in the results. Furthermore, due to the study’s cross-sectional nature, there were challenges in defining chronological sequence, making it difficult to establish a causal relationship [25]. Moreover, there is a possibility of recall bias. For example, many caregivers may not have remembered their children’s diarrhoeal illness in the previous two weeks.

## Conclusion

Childhood diarrhoeal illness in Uganda is still a public health concern, as reflected in the findings reported in this study. The prevalence of diarrhoeal disease was 20.9%, which makes its prevention a priority in this population. In addition, the factors identified that were significantly associated with diarrhoeal illness provide an opportunity to create targeted interventions that will reduce diarrhoeal illness effectively. Uganda has already implemented the WASH program, rotavirus vaccination program as well as other strategies and interventions across the country. The study provided an opportunity to review the interventions that are currently in place to combat diarrhoeal diseases and provide evidence to support the implementation of additional prevention strategies towards averting diarrhoeal illness.

## Data Availability

The datasets generated and/or analysed during the current study are not publicly available due to ethics restrictions, but are available from the corresponding author on reasonable request.

## List of abbreviations

WASH: Water and Sanitation Hygiene
UDHS: Uganda Demographic Health Survey
UBOS: Uganda Bureau Of Statistics
STI: Sexually Transmitted Infections
HIV: Human Immunodeficiency Virus
